# Supplementary material for: HPV infection and the genital cytokine milieu in women at high risk of HIV acquisition
Source: Nat Commun. 2019 Nov 19;10:5227. doi: 10.1038/s41467-019-13089-2 (PMC6863918; doi:10.1038/s41467-019-13089-2)
Supplement: Supplementary file 1 — Supplementary Information [file 41467_2019_13089_MOESM1_ESM.pdf]

Supplementary Information Data

HPV infection and the genital cytokine milieu in women at high risk of HIV acquisition, Liebenberg et al.

Supplementary Figure 1. Sensitivity analysis of HPV infection and genital cytokines in the presence of other current STIs. Comparisons reflect mean differences in cytokine concentrations in women who acquired any HPV type relative to women who remained HPV negative either (a) in the entire cohort, controlling only for STI symptoms and not laboratory detection of STIs (N=494 women; 404 Any Acquired visits and 173 Remained Negative visits), or in a subset of women with laboratory measures of current STI available (N=280 women; 198 Any Acquired visits and 161 Remained Negative events) and either (b) not controlling for current STI, or (c) controlling for current STI. Individual associations are shown between HPV status and pro-inflammatory cytokines (red), chemokines (blue), growth/hematopoietic factors (green), adaptive response cytokines (purple) and regulatory cytokines (orange), with error bars depicting 95% confidence intervals. The models test the hypothesis that the mean cytokine levels in comparator groups are equal i.e. β=0. The dotted line at β=0 distinguishes higher (to the right of the line) from lower (to the left of the line) mean cytokine differences in the respective categories relative to the remained HPV negative group. Grey shadings represent genital cytokines previously associated with HIV risk in this cohort: IP-10, IL-8, MIP-1α and MIP-1β. Filled shapes represent significant associations after FDR adjustment using a threshold of 0.05 in multivariable models controlling for baseline treatment group, age, age of sexual debut, number of sexual partners number of sex acts (past 30 days), presence of STI symptoms, HSV-2 status, condom use, marital status, and whether women were living with regular partners.

Supplementary Table 1. HIV incidence by prevalent HPV type

Supplementary Table 2. Characteristics of women who remained HPV negative, and who acquired, cleared or retained HPV infection

Supplementary Table 3. Tenofovir concentrations and the incidence, clearance and persistence of HPV.

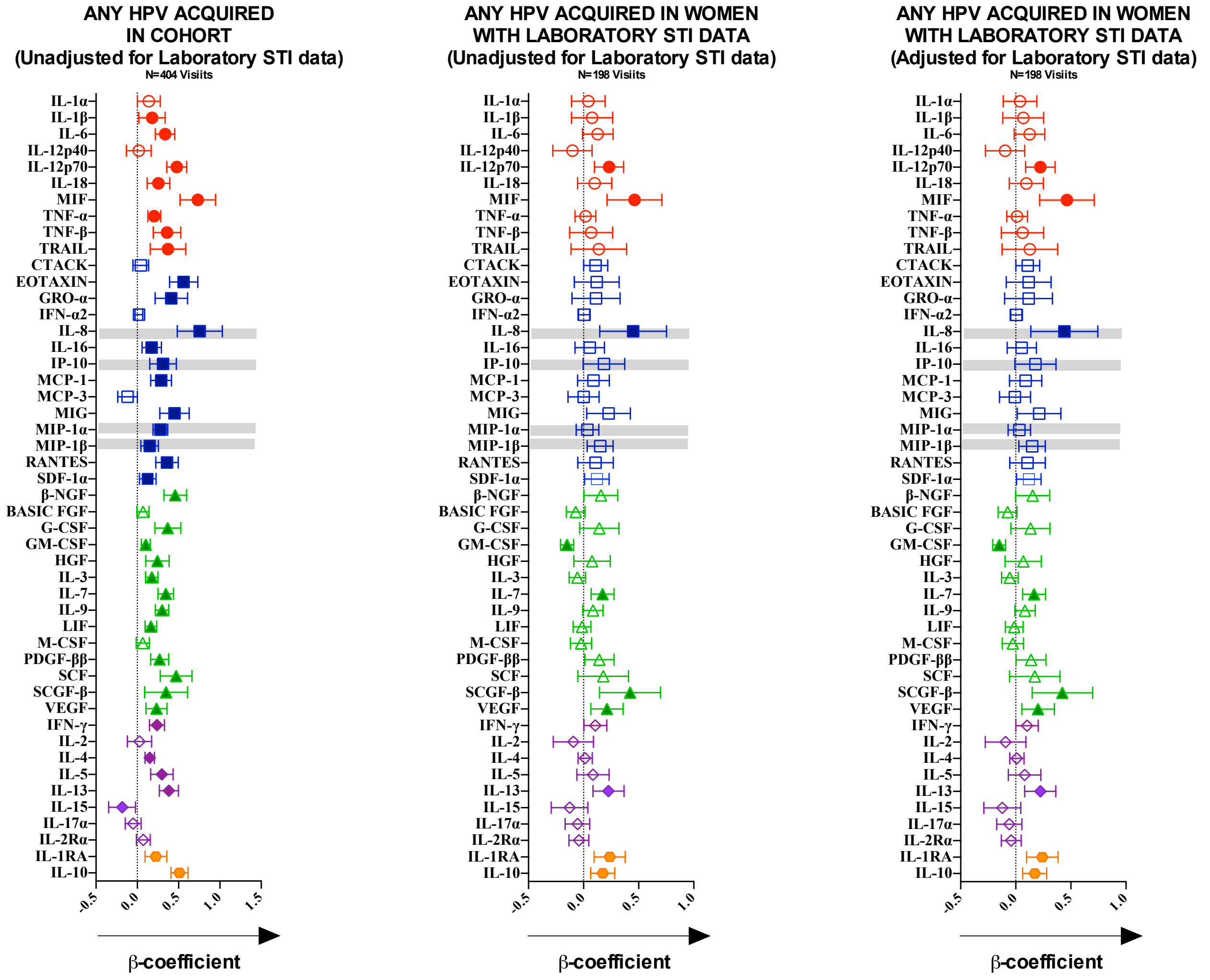

Supplementary Figure 1. Sensitivity analysis of HPV infection and genital cytokines in the presence of other current STIs.

Supplementary Table 1. HIV incidence by prevalent HPV type

	IARC cat#	HPV vaccine types*	Women Years (N)	# HIV Events	HIV IR/100wy (95% CI)	HR (95% CI)	p-value	False Discovery
HPV1S39	2B		27.3 (19)	5	18.3 (5.9-42.7)	10.2 (2.6-39.8)	0.001	0.016
HPV69	2B		27.7 (22)	5	18.1 (5.9-42.2)	5.8 (1.6-21.1)	0.009	0.048
HPV59	1		69.1 (48)	11	15.9 (7.9-28.5)	6.7 (2.4-18.5)	<0.001	0.009
HPV82	2B		40.2 (26)	5	12.4 (4.0-29.0)	2.7 (0.8-9.8)	0.123	0.185
HPV51	1		116.3 (76)	14	12 (6.6-20.2)	4.5 (1.6-12.2)	0.004	0.042
HPV68	2A		61.7 (41)	7	11.3 (4.6-23.4)	4.8 (1.6-14.6)	0.006	0.048
HPV35	1		107.0 (73)	12	11.2 (5.8-19.6)	3.7 (1.4-10.0)	0.009	0.048
HPV11	3	4,9	27.1 (20)	3	11.1 (2.3-32.3)	3.1 (0.7-14.1)	0.137	0.197
HPV71	3		38.5 (27)	4	10.4 (2.8-26.6)	3.6 (0.9-15.2)	0.078	0.147
HPV67	2B		49.7 (31)	5	10.1 (3.3-23.5)	3.7 (1.1-12.4)	0.034	0.112
HPV33	1	9	85.5 (53)	8	9.4 (4.0-18.4)	3.7 (1.3-10.8)	0.017	0.078
HPV66	2B		65.0 (43)	6	9.2 (3.4-20.1)	3.4 (1.0-11.3)	0.05	0.119
HPV61	3		68.0 (46)	6	8.8 (3.2-19.2)	3.5 (1.0-12.2)	0.047	0.119
HPVCP6 108	3		57.3 (40)	5	8.7 (2.8-20.4)	3.6 (1.0-13.0)	0.047	0.119
HPV83	3		58.4 (39)	5	8.6 (2.8-20.0)	3.5 (0.9-13.2)	0.063	0.134
HPV84	3		141.0 (91)	12	8.5 (4.4-14.9)	2.9 (1.1-7.4)	0.028	0.111
HPV16	1	2,4,9	129.7 (84)	11	8.5 (4.2-15.2)	3.1 (1.1-8.5)	0.031	0.111
HPV58	1	9	108.5 (70)	9	8.3 (3.8-15.7)	3.9 (1.4-10.9)	0.009	0.048
HPV55	3		48.3 (31)	4	8.3 (2.3-21.2)	3.2 (0.9-11.7)	0.075	0.147
HPV70	2B		61.3 (41)	5	8.2 (2.7-19.0)	3.4 (1.1-10.8)	0.041	0.119
HPV40	3		25.6 (16)	2	7.8 (0.9-28.3)	5.5 (0.7-43.4)	0.107	0.169
HPV45	1	9	64.9 (43)	5	7.7 (2.5-18.0)	2.9 (0.8-10.0)	0.100	0.169
HPV39	1		66.5 (42)	5	7.5 (2.4-17.5)	3.0 (0.9-10.3)	0.082	0.148
HPV81	3		57.5 (37)	4	7 (1.9-17.8)	3.5 (1.0-12.6)	0.054	0.121
HPV42	3		48.4 (31)	3	6.2 (1.3-18.1)	1.6 (0.3-7.7)	0.546	0.596
HPV06	3	4,9	50.5 (33)	3	5.9 (1.2-17.4)	3.7 (0.8-17.7)	0.108	0.169
HPV56	1		34.5 (23)	2	5.8 (0.7-20.9)	3.0 (0.5-17.3)	0.212	0.282
HPV31	1	9	73.5 (48)	4	5.4 (1.5-13.9)	1.7 (0.4-6.4)	0.449	0.521
HPV52	1	9	74.5 (47)	4	5.4 (1.5-13.7)	2.0 (0.5-7.8)	0.298	0.383
HPV54	3		57.2 (38)	3	5.2 (1.1-15.3)	2.6 (0.6-11.0)	0.209	0.282
HPV72	3		59.7 (40)	3	5.0 (1.0-14.7)	1.6 (0.4-6.7)	0.522	0.587
HPV62	3		121.4 (75)	6	4.9 (1.8-10.8)	1.6 (0.5-4.9)	0.433	0.520
HPV26	2B		25.0 (16)	1	4.0 (0.1-22.3)	0.8 (0.1-6.7)	0.796	0.843
HPV53	2B		78.8 (51)	3	3.8 (0.8-11.1)	1.1 (0.3-4.5)	0.884	0.898
HPV73	2B		41.6 (29)	1	2.4 (0.1-13.4)	1.2 (0.1-11.7)	0.898	0.898
HPV18	1	2,4,9	84.5 (55)	1	1.2 (0.0-6.6)	0.3 (0.0-2.8)	0.312	0.387
HPV64	3		2.8 (2)	0	0 (0.0-129.5)	-	-	-

#IARC (International Agency for Research on Cancer) categories 1, 2, and 3 refer to carcinogenic, probably (2A)/possibly (2B) carcinogenic, and non-carcinogenic HPV respectively. Categories 1 and 2A were included in the study definition of oncogenic types. *Vaccine types 2, 4, 9 refer to bivalent Cervarix®, quadrivalent Gardasil®, and nonavalent Gardasil®9 HPV types respectively. CI, confidence interval.

Supplementary Table 2. Characteristics of women who remained HPV negative, and who acquired, cleared or retained HPV infection

		Mutually-Exclusive Categories			Overlapping Categories		
	Remained negative	Only Acquired	Only Cleared	Only Persisted	Acquired Any	Cleared Any	Persisted Any
Median age (IQR), years	25.5 (21 - 31)	22 (20 - 27)**	24 (21 - 29)	24 (20 - 28)	22 (20 - 25)**	22 (20 - 26)***	22 (20 - 26)***
Median age sex debut (IQR), years	17 (16 - 19)	17 (16 - 19)	17 (16 - 18)	17 (16 - 18)	17 (16 - 18)	17 (16 - 18)	17 (16 - 19)
Median lifetime partners (IQR), years	2 (1 - 3)	2 (1 - 3)	2 (1 - 3)	2 (1 - 3)	2 (1 - 3)	2 (1 - 3)	2 (1 - 3)
Median number of sex acts in last 30 days (IQR), years	6.5 (4 - 12)	6 (4 - 10)	6 (4 - 10)	8 (4 - 10)	6 (3 - 10)	6 (3 - 10)*	6 (3 - 10)
Contraception n, (%)							
Injectable	90 (83.3)	72 (84.7)	103 (79.2)	29 (87.9)	346 (85.4)**	396 (83.0)	207 (87.7)
Oral	12 (11.1)	12 (14.1)	21 (16.2)	3 (9.1)	56 (13.8)	71 (14.9)	26 (11.0)
Other	6 (5.6)	1 (1.2)	6 (4.6)	1 (3.0)	3 (0.7)	10 (2.1)	3 (1.3)
Condom use n, (%)							
Always	30 (27.8)	26 (30.6)	35 (26.9)	13 (39.4)	118 (29.1)**	136 (28.5)*	63 (26.7)**
Most Times	15 (13.9)	13 (15.3)	19 (14.6)	4 (12.1)	78 (19.3)	89 (18.7)	52 (22.0)
Occasionally	34 (31.5)	31 (36.5)	51 (39.2)	10 (30.3)	156 (38.5)	184 (38.6)	93 (39.4)
Never	29 (26.9)	15 (17.6)	25 (19.2)	6 (18.2)	53 (13.1)	68 (14.3)	28 (11.9)
Marital status n, (%)							
Married	18 (16.7)	4 (4.7)*	8 (6.2)*	3 (9.1)	10 (2.5)***	19 (4.0)***	10 (4.2)***
Stable	81 (75.0)	72 (84.7)	116 (89.2)	29 (87.9)	368 (90.9)	434 (91.0)	213 (90.3)
Other	9 (8.3)	9 (10.6)	6 (4.6)	1 (3.0)	27 (6.7)	24 (5.0)	13 (5.5)
Living with regular partner n, (%)							
Yes	33 (30.6)	4 (4.7)***	20 (15.4)**	7 (21.2)	22 (5.4)***	47 (9.9)***	27 (11.4)***
No	72 (66.7)	78 (91.8)	110 (84.6)	26 (78.8)	380 (93.8)	430 (90.1)	209 (88.6)
Baseline STI symptoms n, (%)							
No	68 (63.0)	58 (68.2)	82 (63.1)	21 (63.6)	257 (63.5)	298 (62.5)	141 (59.7)
Yes	40 (37.0)	27 (31.8)	48 (36.9)	12 (36.4)	148 (36.5)	179 (37.5)	95 (40.3)
HSV-2 serology n, (%)							
Positive	68 (63.0)	42 (49.4)	69 (53.5)	20 (60.6)	193 (47.7)*	235 (49.4)	125 (53.0)
Negative	40 (37.0)	41 (48.2)	60 (46.5)	13 (39.4)	205 (50.6)	235 (49.4)	107 (45.3)
Sex partner has other partners n, (%)							
Yes	26 (24.3)	14 (17.1)	27 (21.3)	4 (12.1)	87 (22.1)	105 (22.6)*	51 (22.6)
No	26 (24.3)	16 (19.5)	19 (15.0)	5 (15.2)	63 (16.0)	67 (14.4)	34 (15.0)
Don't know	55 (51.4)	52 (63.4)	81 (63.8)	24 (72.7)	244 (61.9)	292 (62.9)	141 (62.4)

* p<0.05; ** p<0.01; *** p<0.001. Factors associated with HPV were included in multivariate models assessing the relationship between HIV and/or cytokines, and HPV. IQR, Interquartile range).

Supplementary Table 3. Tenofovir concentrations and the incidence, clearance and persistence of HPV.

	Placebo (N=387)	< 1000 ng/ml TFV (N =204)	≥ 1000 ng/ml TFV (N =95)	p-value
Only Acquired HPV (%, n)	11.1 (41)	10.1 (20)	10.8 (10)	0.937
Acquired any HPV (%, n)	55.4 (204)	54.6 (108)	50.5 (47)	0.698
Only Cleared HPV (%, n)	17.4 (64)	15.7 (31)	25.8 (24)	0.105
Cleared any HPV (%, n)	65.8 (242)	64.1 (127)	71.0 (66)	0.516
Only Persisted HPV (%, n)	3.5 (13)	6.6 (13)	2.2 (2)	0.161
Persisted any HPV (%, n)	32.3 (119)	35.9 (71)	29.0 (27)	0.492
