# Supplementary material for: HPV infection and the genital cytokine milieu in women at high risk of HIV acquisition
Source: Nat Commun. 2019 Nov 19;10:5227. doi: 10.1038/s41467-019-13089-2 (PMC6863918; doi:10.1038/s41467-019-13089-2)
Supplement: Supplementary file 3 — Reporting Summary [file 41467_2019_13089_MOESM3_ESM.pdf]

nature research

Corresponding author(s): Lenine Liebenberg

Last updated by author(s): Oct 17, 2019

Reporting Summary

Nature Research wishes to improve the reproducibility of the work that we publish. This form provides structure for consistency and transparency in reporting. For further information on Nature Research policies, see Authors & Referees and the Editorial Policy Checklist.

Statistics

For all statistical analyses, confirm that the following items are present in the figure legend, table legend, main text, or Methods section.

n/a	Confirmed	
☐	☒	The exact sample size (*n*) for each experimental group/condition, given as a discrete number and unit of measurement
☐	☒	A statement on whether measurements were taken from distinct samples or whether the same sample was measured repeatedly
☐	☒	The statistical test(s) used AND whether they are one- or two-sided<br>*Only common tests should be described solely by name; describe more complex techniques in the Methods section.*
☐	☒	A description of all covariates tested
☐	☒	A description of any assumptions or corrections, such as tests of normality and adjustment for multiple comparisons
☐	☒	A full description of the statistical parameters including central tendency (e.g. means) or other basic estimates (e.g. regression coefficient) AND variation (e.g. standard deviation) or associated estimates of uncertainty (e.g. confidence intervals)
☒	☐	For null hypothesis testing, the test statistic (e.g. *F*, *t*, *r*) with confidence intervals, effect sizes, degrees of freedom and *P* value noted<br>*Give P values as exact values whenever suitable.*
☒	☐	For Bayesian analysis, information on the choice of priors and Markov chain Monte Carlo settings
☐	☒	For hierarchical and complex designs, identification of the appropriate level for tests and full reporting of outcomes
☐	☒	Estimates of effect sizes (e.g. Cohen's *d*, Pearson's *r*), indicating how they were calculated

Our web collection on statistics for biologists contains articles on many of the points above.

Software and code

Policy information about availability of computer code

Data collection	MagNA pure LC 2.0 instrument software, Bio-Plex Manager software version 6, SPSS version 24, SAS version 9.3, and Microsoft Excel 14.7.2
Data analysis	Bio-Plex Manager software version 6, SPSS version 24, SAS version 9.3, and Microsoft Excel 14.7.2

For manuscripts utilizing custom algorithms or software that are central to the research but not yet described in published literature, software must be made available to editors/reviewers. We strongly encourage code deposition in a community repository (e.g. GitHub). See the Nature Research guidelines for submitting code & software for further information.

Data

Policy information about availability of data

All manuscripts must include a data availability statement. This statement should provide the following information, where applicable:

- Accession codes, unique identifiers, or web links for publicly available datasets
- A list of figures that have associated raw data
- A description of any restrictions on data availability

The source data underlying Figures 2, 4, 5, 6, and Supplementary Figure 1 are provided in this Source Data file. Any other data will be made available through request on a dedicated portal on the CAPRISA website (http://www.caprisa.org/).

Field-specific reporting

Please select the one below that is the best fit for your research. If you are not sure, read the appropriate sections before making your selection.

☒ Life sciences ☐ Behavioural & social sciences ☐ Ecological, evolutionary & environmental sciences

For a reference copy of the document with all sections, see nature.com/documents/nr-reporting-summary-flat.pdf

Life sciences study design

All studies must disclose on these points even when the disclosure is negative.

Sample size	Available genital specimens from all time points of all participants in the CAPRISA 004 trial were included in this analysis. The original cohort was powered to detect the effectiveness of tenofovir 1% gel, as previously described (Abdool Karim et al., Science 2010).
Data exclusions	Participant specimens for the original trial were not available for 13% of participants. We were not able to carry out analyses in instances where storage consent was not provided, no specimens were available, or when participants acquired HIV before samples could be obtained.
Replication	Clinical specimens were analyzed. A portion were analyzed in duplicate to calculate intra-plate variability and these data are available if required. The validated HPV genotyping data is available on request.
Randomization	Samples were analyzed randomly, with plate design for HPV DNA and cytokine measurements done without any knowledge of clinical or outcome variables.
Blinding	Laboratory personnel were blinded to all clinical and epidemiological data, and were only given access (where applicable) once the final validated data was locked.

Reporting for specific materials, systems and methods

We require information from authors about some types of materials, experimental systems and methods used in many studies. Here, indicate whether each material, system or method listed is relevant to your study. If you are not sure if a list item applies to your research, read the appropriate section before selecting a response.

Materials & experimental systems

n/a	Involved in the study	
☐	☒	Antibodies
☒	☐	Eukaryotic cell lines
☒	☐	Palaeontology
☒	☐	Animals and other organisms
☐	☒	Human research participants
☐	☒	Clinical data

Methods

n/a	Involved in the study	
☒	☐	ChIP-seq
☒	☐	Flow cytometry
☒	☐	MRI-based neuroimaging

Antibodies

Antibodies used	Anti-cytokine capture antibodies and anti-cytokine biotinylated detection antibodies (Bio-Rad 27-plex and 21-Plex Cytokine, Chemokine and Growth Factor Assays)
Validation	All antibodies used in Multiplex ELISA assays were validated by the manufacturer (BioRad).

Human research participants

Policy information about studies involving human research participants

Population characteristics	All participants were women enrolled in the CAPRISA 004 clinical trial that tested the safety and efficacy of TFV 1% Gel (Abdool Karim et al., Science 2010; Karim et al., Trials 2011). We have included analysis of the impact of human papillomavirus (HPV) infection status on HIV acquisition risk, and of the association between HPV status and genital cytokines. A number of co-variates collected during the clinical trial have been used to adjust multi-variable analyses; most of these relate to risk factors for HIV acquisition, prevalent HPV infection, and/or potential correlates of mucosal cytokine levels.
Recruitment	The study involved a retrospective analysis of HPV infection, HIV infection and genital cytokine concentrations in available genital specimens from all participants enrolled in the CAPRISA 004 clinical trial. Recruitment of the original population is detailed in Abdool Karim et al., Science 2010; Karim et al., Trials 2011.
Ethics oversight	The trial (NCT00441298) and this secondary analysis was approved by the University of KwaZulu-Natal's Biomedical Research

Ethics oversight	Ethics Committee (E111/06), Family Health International's Protection of Human Subjects Committee (#9946) and the South African Medicines Control Council (#20060835)

Note that full information on the approval of the study protocol must also be provided in the manuscript.

Clinical data

Policy information about clinical studies

All manuscripts should comply with the ICMJE guidelines for publication of clinical research and a completed CONSORT checklist must be included with all submissions.

Clinical trial registration	The parent clinical trial was registered as NCT00441298
Study protocol	The protocol for the parent clinical trial can be accessed at: https://www.caprisa.org/DBFile/Files/d97acdd9-5531-4d3d-a522-8d1718e040dd/CAPRISA%20004%20-%20Tenofovir%20gel%20trial.pdf
Data collection	Data collection details for the parent study can be accessed from Abdool Karim et al., 2010 or from the study protocol: https://www.caprisa.org/DBFile/Files/d97acdd9-5531-4d3d-a522-8d1718e040dd/CAPRISA%20004%20-%20Tenofovir%20gel%20trial.pdf. In this study, HPV genotyping and genital cytokine data were collected from cervicovaginal lavage specimens collected at months 3, 12, 24 and at study exit from all participants, where exit could fall anytime between month 1 and month 30.
Outcomes	Study outcomes for the parent study can be accessed from Abdool Karim et al., 2010 or from the study protocol: https://www.caprisa.org/DBFile/Files/d97acdd9-5531-4d3d-a522-8d1718e040dd/CAPRISA%20004%20-%20Tenofovir%20gel%20trial.pdf. In this study, we assessed the association between HPV status and HIV risk, and the relationship between HPV status and genital cytokine concentrations in participants of the CAPRISA 004 clinical trial.
